# A Comparative Metagenomics Study on Gastrointestinal Microbiota in Amphibious Mudskippers and Other Vertebrate Animals

**DOI:** 10.3390/ani9090660

**Published:** 2019-09-06

**Authors:** Yunhai Yi, Lifeng Liang, Zhilin Wang, Peng Ai, Xinxin You, Chao Bian, Qiong Shi, Bo Dong

**Affiliations:** 1 Agro-Biological Gene Research Center, Guangdong Academy of Agricultural Sciences, Guangzhou 510006, China (Y.Y.) (Z.W.); 2 BGI Education Center, University of Chinese Academy of Sciences, Shenzhen 518083, China; 3 Shenzhen Key Lab of Marine Genomics, Guangdong Provincial Key Lab of Molecular Breeding in Marine Economic Animals, BGI Academy of Marine Sciences, BGI Marine, BGI, Shenzhen 518083, China (L.L.) (C.B.); 4 Research & Development Department, Guangzhou Genedenovo Biotechnology Co. Ltd., Guangzhou 510320, China

**Keywords:** microbiome, metagenome sequencing, 16S ribosomal DNA (rDNA), operational taxonomic unit (OTU), bacteriocin-related genes, terrestrial adaptation, symbiotic bacteria

## Abstract

**Simple Summary:**

Mudskippers are important ecological components of intertidal ecosystems. It was proposed that their guts may play significant roles for terrestrial adaptations of these amphibious fishes. However, their gastrointestinal components and differences in microbiota with other vertebrates were never reported. Here, we performed a comparative metagenome analysis among various vertebrate groups, classified by living habitats and feeding habits, and also acquired microbial gene catalogs of five common fish species. Our findings confirmed the dominant microbial genera in each vertebrate group, as well as bacteriocin-related genes in the five common fish species, for discussion of their relationships with fish pathogenic diseases. Our big data will support in-depth investigations into potential roles of gastrointestinal microbiota to hosts and related applications in aquaculture practices.

**Abstract:**

Gut microbiomes in various fish species were widely investigated with the rapid development of next-generation sequencing technologies. However, little is known about gastrointestinal (GI) microbial communities in mudskippers, a representative group of marine amphibious fishes, and their comparisons with other vertebrate animals from different habitats. Here, we performed a comprehensive analysis on microbial composition in five representative vertebrate groups (including amphibious mudskippers, marine and freshwater aquatic fishes, amphibians, and terrestrial animals) via operational taxonomic unit (OTU) survey and obtained a microbial gene catalog of five common fish species by metagenome sequencing. We observed that Cyanobacteria, Proteobacteria, Firmicutes, Bacteroidetes, and Fusobacteria were the most substantial bacteria in mudskippers. Differential variances in composition patterns of GI microbiota among the vertebrate groups were determined, although Proteobacteria and Firmicutes were the shared phyla with high abundance. In addition, *Cetobacterium* and *Photobacterium* were the most abundant genera in core OTUs of these examined omnivores, carnivores, and herbivores. Our metagenomic analysis also showed significant differences between the representative blue-spotted mudskipper and grass carp (both are herbivorous fishes) in microbes at the phylum and class levels and functional gene terms. Moreover, several bacteriocin-related genes were identified in the five common fishes, suggesting their potential contributions to pathogen resistance. In summary, our present work not only sheds new light on the correlation of GI microbiota composition with living habitats and feeding habits of the hosts, but also provides valuable bacterial genetic resources for healthy growth of aquaculture fishes.

## 1. Introduction

Metagenomics developed rapidly with the advance of next-generation sequencing technologies; however, related researches on fish species are lagging behind [1]. Fish gut microbiota has a profound impact on the host physiology. Several economically important fish species, such as tilapias, Atlantic salmon, and carps, were studied [2]. It was long proposed that the fish genetic background and living niche select for a “core microbiota” to maintain some essential functions [3]. These bacteria co-evolve with the hosts and compete for the common resources in gastrointestinal (GI) tract over million years. They maintain many physiological functions in fishes, such as the antagonism of pathogens, the proliferation of enteric epithelium, and the maturation of immunity [4,5]. 

Amphibious fishes, such as mudskippers, are interesting groups of vertebrates that can thrive in inshore seawater, as well as on land, offering useful models for studying the genetic changes associated with the evolutionary water-to-land transition of vertebrates [6]. It was proposed that the gut may play a significant role in aerial gaseous exchange and restriction of water loss during terrestrial adaptations of amphibious fishes [7]. Mudskippers are valuable ecological components of intertidal mudflats, which are complex and dynamic conditions. However, little is known about their GI microbiota, the second genome set within their bodies. Comparisons of the GI microbiome among various vertebrates from diverse niches to elaborate differences in their distinct immune systems or traits are also scarce [8]. 

Here, we employed the routine 16S amplicon and metagenome sequencing to compare the differential composition of GI microbiota between mudskippers and other vertebrate animals, including aquatic fishes and terrestrial vertebrates. A comprehensive examination of what microbial communities are shared by all the examined vertebrates and what microbes are specific to the living habitats and feeding habits was also investigated. Furthermore, bacteriocin-related genes were analyzed in the sequenced metagenomes for potential immunological functions. Identification of other natural products or gene clusters for secondary metabolites was also facilitated by this genome mining approach and genetic resource. These findings will provide basic genetic support for in-depth investigations into fish microbiomes and elucidation of microbial diversity in vertebrates.

## 2. Materials and Methods

### 2.1. Sample Collection

In this study, seven fish species were collected from a local area within the Guangdong Province of China. These fishes include pond-cultured grass carp (*Ctenopharyngodon idellus*, CI; *n* = 10) and channel catfish (*Ietalurus punetaus*, IP; *n* = 10) from the Pearl River Fisheries Research Institute (Guangzhou, Guangdong, China), wild-caught blue-spotted mudskipper (*Boleophthalmus pectinirostris*, BP; *n* = 13) and giant-fin mudskipper (*Periophthalmus magnuspinnatus*, PM; *n* = 1) from Island Qi’ao (Zhuhai, Guangdong, China), and cage-cultured orange-spotted grouper (*Epinephelus coioides*, EC; *n* = 3), lined seahorse (*Hippocampus erectus*, HE; *n* = 2), and golden rabbitfish (*Siganus guttatus*, SG; *n* = 6) from the Baguang Aquaculture Base of BGI Marine (Shenzhen, Guangdong, China). Individuals with an average body weight of 100–200 g were selected to obtain gastrointestinal contents.

The body surface of each fish was rinsed with sterile distilled water and then 70% ethanol to reduce contamination. The GI tract was dissected aseptically from the abdominal cavity, and the GI contents were squeezed out for separate harvest. All samples were immediately stored at −80 °C before use.

All experiments were performed in accordance with the guidelines of the Animal Ethics Committee and approved by the Institutional Review Board on Bioethics and Biosafety of BGI, China (No. FT15103).

### 2.2. Microbial Amplicon Sequencing and Data Analysis

Microbial DNA was extracted from the 45 samples using an E.Z.N.A. stool DNA Kit (Omega Biotek, Norcross, GA, USA) according to the manufacturer’s instructions. The 16S rDNA V3–V4 region of each ribosomal DNA gene was amplified by PCR using a standard primer pair (341 forward (F): CCTACGGGNGGCWGCAG; 806 reverse ®: GGACTACHVGGGTAT CTAAT). Amplicons were extracted from 2% agarose gels for subsequent purification with an AxyPrep DNA Gel Extraction Kit (Axygen Biosciences, Union City, CA, USA) and quantification using QuantiFluor-ST (Promega, Madison, WI, USA). Purified amplicons were then pooled in equal molar for paired-end sequencing (2 × 250 bp) in an Illumina Hiseq2500 platform (San Diego, CA, USA). 

For the main purpose of comparing amphibious mudskippers with other vertebrates from different habitats, we downloaded 91 datasets of 16S rDNA V3–V4 amplicon sequencing from the GI of various vertebrate species (NCBI accession numbers are listed in Table S1, Supplementary Materials). In total, 136 samples were divided into five groups, including amphibious mudskippers (MU; *n* = 14), freshwater (FR; *n* = 54) and marine (MA; *n* = 23) aquatic fishes, amphibians (AM; *n* = 25), and terrestrial animals (TE; *n* = 20). On the basis of their feeding habits, they were also categorized into herbivorous (HE; *n* = 27), carnivorous (CA; *n* = 20), and omnivorous (OM; *n* = 89) categories.

Raw reads with adapter sequences and low-quality reads were removed. These filtered clean reads were then merged as raw tags using FLASH (v 1.2.11) [9] with a minimum overlap of 10 bp and mismatch error rates less than 2%. QIIME (v 1.9.1) pipeline [10,11] was employed to filter noisy sequences to generate high-quality clean tags, which were clustered into operational taxonomic units (OTUs) by USEARCH (v 7.0.1090) [11]. Effective tags were clustered into OTUs with over 97% similarity using the UPARSE pipeline [12]. Reference-based chimera checking was performed by UCHIME (v 4.2.40) [13]. All tags were finally mapped to each OTU representative sequence using USEARCH GLOBAL [14], and then the tag number of each OTU in every sample was summarized for quantification of OTU abundance. The tag sequence with the highest abundance was selected as the representative one within each cluster, which was subsequently classified into organisms by a naive Bayesian model using the RDP classifier (v 2.2) [15] based on Greengenes database [16]. Alpha diversity was analyzed in the QIIME. Finally, functional prediction of the OTUs was inferred using Tax4Fun (v 1.0) [17]. Metastats (http://metastats.cbcb.umd.edu/) and R (v3.1.1; https://www.r-project.org/) were used to determine the taxonomic groups with significant differences between the sample groups. 

### 2.3. Metagenome Sequencing and Analysis

Based on the sampling conditions, 19 metagenome DNA samples collected from five common fish species (BP, *n* = 4; PM, *n* = 1; CI, *n* = 10; IP, *n* = 2; SG, *n* = 2) were further processed into qualified libraries and then sequenced via an Illumina Hiseq2500 platform. Qualified sequencing data were subsequently preprocessed to remove the host contamination and assembled de novo with SOAPdenovo2 [18] and Rabbit [19]. MetaGeneMark (v 2.10) [20] were employed to predict genes from the assembled contigs. Genes from different samples were combined and clustered using CD-Hit [21]. The integrated gene catalogs were used to BLAST (Basic Local Alignment Search Tool; National Center for Biotechnology Information, Bethesda, MD, USA)_against several public databases, including NCBI non-redundant (Nr), Swiss-Prot, COG (Cluster of Orthologous Groups), KEGG (Kyoto Encyclopedia of Genes and Genomes), GO (Gene Ontology), CAZy (Carbohydrate-Active Enzymes), EggNOG (Evolutionary Genealogy of Genes: Non-Supervised Orthologous Groups), and ARDB (Antibiotic Resistance Genes Database), to obtain functional annotations (with E ≤ 1.0 × 10^−5^). Then all hit results were assigned to the corresponding NCBI taxonomy using MEGAN (v 4.6) [22], and the relative abundance profile of each taxonomy level in the examined samples was quantified. Calculation of the relative gene abundance was realized by Pathoscope (v 1.0) [23]. For identification of differentially expressed genes (DEGs), we performed principle component analysis (PCA), GO enrichment, and pathway enrichment analysis.

The datasets generated for this study, including the complete 16S rDNA gene amplicon sequencing data of the 45 samples and metagenome of the 19 samples, are available in the China National GeneBank Database (CNGBdb) under the project identifier (ID) CNP0000345. Detailed accession numbers of these datasets are listed in Table S1 (Supplementary Materials).

### 2.4. Statistical Analysis

In order to examine the similarities and differences of OTU compositions in the samples of different groups, PCA and non-metric multi-dimensional scaling (NMDS) analyses of the OTU relative abundance values were used to construct a two-dimensional (2D) graph to summarize affecting factors and to simplify the data complexity. Compared with PCA, NMDS can minimize dimensions and preserve distance between data points. Differences among multiple groups were calculated by a Kruskal–Wallis test [24], and *p*-values less than 0.05 were retained for significant difference calculation by the Wilcoxon rank-sum test [25]. False discovery rate (FDR) was also used to assess the significance of differences. A box and violin plot of alpha diversity was drawn using the software R (v3.1.1), and Venn diagrams were generated by VennDiagram in the software R.

## 3. Results

### 3.1. Summary of the Achieved 16S Amplicon Data

A total of 9066 OTUs were identified from the pooled 136 samples (see more details in Section 2.2), in which 3736 OTUs were collected from our sequenced 45 samples in this study (see Section 2.1). Detailed information about amplicon and metagenomic sequencing is listed in Table S1 (Supplementary Materials). OTU abundance is summarized in Table S2 (Supplementary Materials). Alpha diversity was applied to analyze the complexity of species diversity in each sample through several indices, including Observed Species, Chao1, Ace, Shannon, and Simpson (see more details in Table S3, Supplementary Materials). There were significant differences among the examined five vertebrate groups.

**Figure 1:**
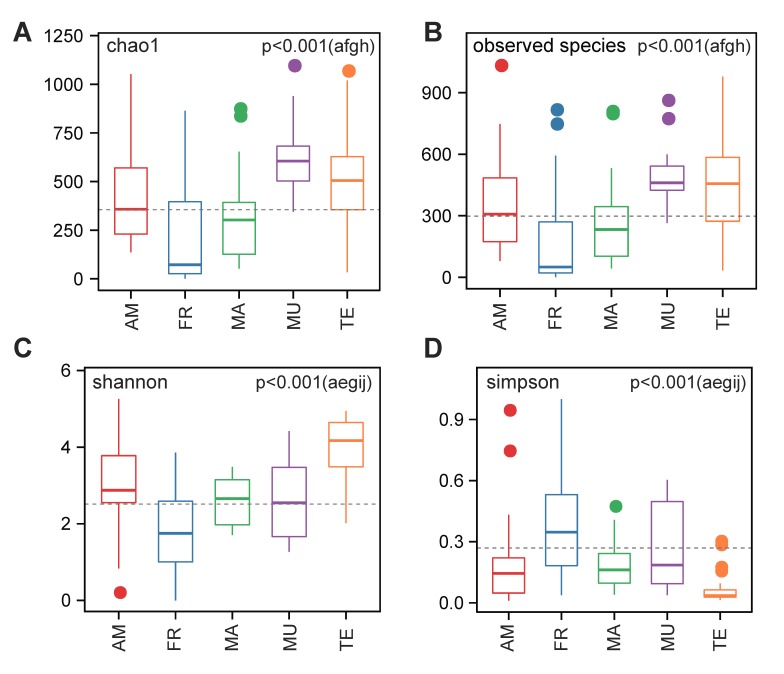
Alpha diversity assessed by richness (**A**,**B**) and diversity (**C**,**D**). Boxes represent the interquartile ranges, lines indicate medians, and whiskers indicate the ranges. The five vertebrate groups (*n* = 136), including amphibious mudskippers (MU; *n* = 14), amphibians (AM; *n* = 25), freshwater fishes (FR; *n* = 54), marine fishes (MA; *n* = 23), and terrestrial animals (TE; *n* = 20), were divided according to their living habitats. a: AM/FR; b: AM/MA; c: AM/MU; d: AM/TE; e: FR/MA; f: FR/MU; g: FR/TE; h: MA/MU; i: MA/TE; j: MU/TE. Please note for the Simpson analysis (**D**) that low values stand for high species diversity.

We observed the lowest species richness in the freshwater fish species and the highest in mudskippers (via Chao1 and Observed Species; see Figure 1A,B). Differences were insignificant either between amphibians and marine/terrestrial species, or between mudskippers and terrestrial species (*p* > 0.05). Similarly, diversity was determined to be the lowest in the freshwater fishes (via Shannon and Simpson); however, terrestrial species had the highest species diversity in this study, and mudskippers did not show significant differences with other groups except the terrestrial animals (see more details in Figure 1C,D). Obviously, our results revealed that the GI microbial communities in vertebrates from different living habitats varied in richness and diversity.

### 3.2. Differential OTUs among the Five Examined Groups

**Figure 2:**
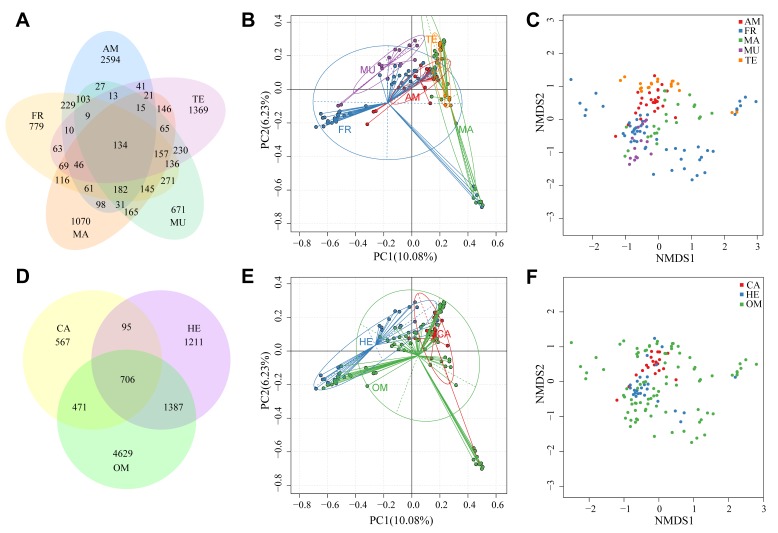
An operational taxonomic unit (out) analysis among the five vertebrate groups. (**A**) Venn diagram displaying the numbers of common and unique OTUs; (**B**) principal component analysis (PCA) diagram and (**C**) non-metric multi-dimensional scaling (NMDS) analyses were employed to distinguish the examined five groups with different living habitats; (**D**) Venn diagram displaying the numbers of common and unique OTUs among the three animal groups with different feeding habits; (**E**) PCA diagram and (**F**) NMDS analyses were used to separate them. OM, omnivore; HE, herbivore; CA: carnivore.

Core microbiomes in the examined vertebrate GIs were obtained after combination of all the OTU taxonomy results (Figure 2A, Table S2, Supplementary Materials). We identified 134 common OTUs among the examined five vertebrate groups with various living habitats. The most abundant sequences referred to the genus *Pelomonas*, under the Comamonadaceae family of the Betaproteobacteria class. However, they were mainly possessed by groupers and the yellowtail scad (*Atule mate*). Interestingly, all the top 23 abundant sequences belonged to Proteobacteria and Firmicutes.

Among the 671 OTUs specific in mudskipper GIs, 39 were classified into the order Rickettsiales with the most abundant sequences, which are endosymbionts of eukaryotic cells proposed to be the sister-group of mitochondria. *Mycoplasma*, lacking cell walls around their cell membranes and naturally resistant to many popular antibiotics, were widely present in mudskippers. As a group of anaerobic bacteria reducing sulfates to sulfides for energy supply, the genus *Desulfobulbus* also had rather high levels in mudskippers, while it was undetectable in other vertebrates. Meanwhile, widespread existence of OTUs in the class of Flavobacteriia and in the family of Oceanospirillaceae should be noted for the mudskippers, since they have several known members (such as *Flavobacterium gelidilacus*) with high pathogenicity in freshwater and/or salt environments [26].

Based on the OTU relative abundance, we performed principal component analysis (PCA) and non-metric multi-dimensional scaling (NMDS) analyses to distinguish microbial composition differences among the sequenced samples (Table S2, Supplementary Materials; Figure 2B–F). In general, different samples were remarkably separated. Within samples in each group, most of them were more closely localized than those out of the group. Interestingly, for the freshwater fishes, we determined a broad range of microbiota composition (Figure 2B,C), almost covering the areas for amphibious and marine fishes. However, as for mudskippers and terrestrial animals, the least variability was presented, possibly implying their restricted adaptation to environments.

For the species divided by different feeding habits, we identified 706 core microbiomes (Figure 2D). The most abundant sequences referred to *Photobacterium damselae*, and they mainly existed in mudskippers, golden rabbitfish, and groupers (Table S2, Supplementary Materials). The fact that this bacterium can cause diseases to fishes should be emphasized. The microbiota community structure of these three groups classified by their feeding habits was clearly separated (Figure 2E,F). Interestingly, GI microbiomes in omnivorous species spanned a wider range than the sum of both herbivores and carnivores; Proteobacteria constituted the largest part in the common microbiota of the three animal groups with various feeding habits.

### 3.3. Results of a Taxonomic Composition Analysis

**Figure 3:**
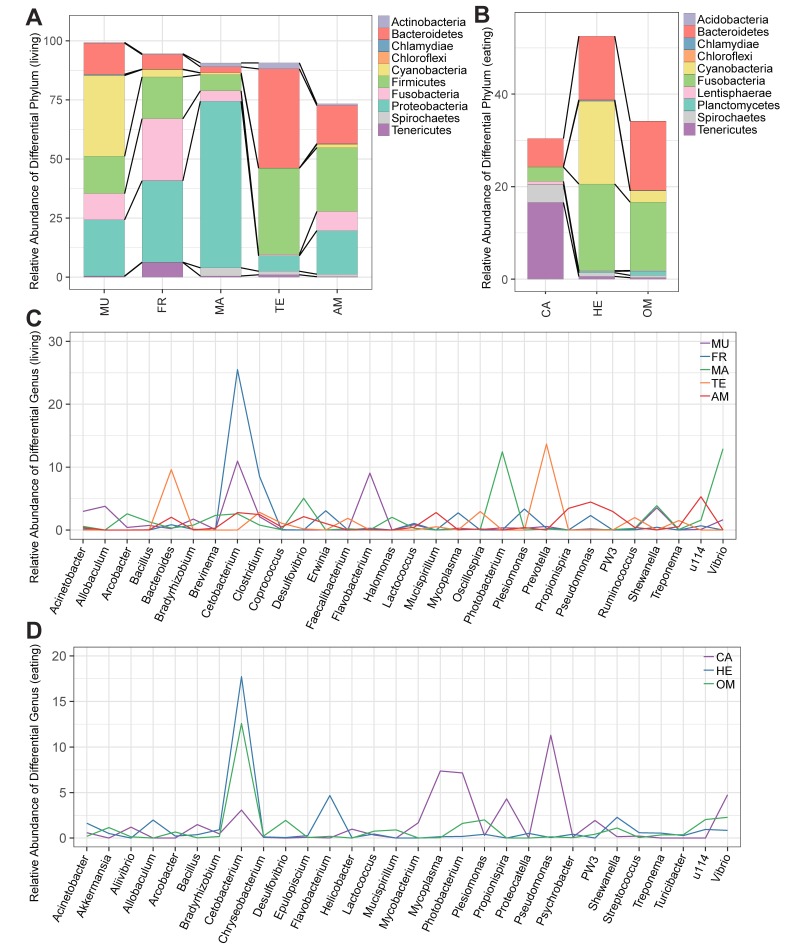
Relative abundance of taxonomic compositions among the examined groups. (**A**) The top 10 abundant phyla in mudskippers (MU) in comparison with other four groups with different living habitats (AM: amphibians, FR: freshwater fishes, MA: marine fishes, TE: terrestrial animals); (**B**) the top 10 abundant phyla in carnivorous (CA) species in comparison with herbivorous (HE) and omnivorous (OM) groups; (**C**) the top 30 abundant genera in mudskippers and (**D**) in herbivorous species in comparison with other groups.

Relative abundance of each taxonomic rank (including phylum, class, order, family, genus, and species) in every sample and the related differential analysis among various groups are summarized in Figure 3. The ratio of each bacterium in any sample is listed in Table S4 (Supplementary Materials). The significant differences among groups were also analyzed, and the statistics are provided in Table S5 (Supplementary Materials) for comparisons of taxonomic composition. In general, the two classification methods indicated that some core microbiomes were correlated with different living conditions or feeding habits while some were not.

The top 10 phyla in mudskippers are summarized in Figure 3A, which demonstrated a different composition pattern from other groups. Cyanobacteria had a rather higher abundance in mudskippers (over 34%) than any other groups. Proteobacteria, Firmicutes, and Bacteroidetes were at relative high levels in all the examined five groups, while Fusobacteria only had an extremely low abundance in the terrestrial animals. In addition, Tenericutes occupied a large proportion in the GI microbiota of the freshwater fishes. Notably, several top phyla in amphibians were at rather low levels in other four groups, such as Synergistetes, Deferribacteres, Verrucomicrobia, and Planctomycetes. When employing the classification of feeding habits (Figure 3B), we found that Synergistetes and Deferribacteres only presented high levels in the omnivorous group (Table S5, Supplementary Materials). Tenericutes and Spirochaetes obviously had a larger proportion in the carnivores than in the herbivores and the omnivores (Figure 3B), while Cyanobacteria were almost undetectable in the carnivores, although it was the second largest part in the herbivores. Bacteroidetes and Fusobacteria in the carnivores were comparatively a bit lower than in the herbivorous and the omnivorous groups. 

The top 30 genera in mudskippers and their relative abundance distribution in the other four groups revealed the diversity of GI microorganisms (Figure 3C). Genus *Cetobacterium* had a rather high abundance in the freshwater fishes, more than double of those in mudskippers. Interestingly, *Flavobacterium* was unusually high in mudskippers. *Vibrio* and *Photobacterium* were dominant genera in the marine fishes, while *Prevotella* and *Bacteroides* prevailed in the terrestrial animals, and *Pseudomonas* was popular in amphibians. Meanwhile, *Propionispira*, *Mycobacterium*, and *Pseudomonas* were among the top 10 genera in carnivorous group, while they were seldom identified in the other two groups (Table S5, Supplementary Materials). *Mycoplasma* and *Photobacterium* were also only rich in the carnivorous species (Figure 3D). *Cetobacterium* was the largest genus both in the herbivorous and the omnivorous groups, while *Flavobacterium* was only popular in the herbivores.

### 3.4. Metagenomic Analysis of Five Common Fish Species

We obtained 19 qualified samples (see more details in Section 2.3) in five common fish species (blue-spotted mudskipper, BP; giant-fin mudskipper, PM; grass carp, CI; channel catfish, IP; and golden rabbitfish, SG) and generated 1.59 billion clean reads, with an entire catalog of 155,217 non-redundant (Nr) genes. A total of 132,183 genes were annotated by the NCBI Nr database, and the others were from public GO, COG, KEGG, and SwissProt databases (see Section 2.3 and Table S6, Supplementary Materials). Finally, these assembled genes were assigned to 4966 bacteria species, 1453 genera, 378 families, 178 orders, 76 classes, and 54 phyla (Table S6, Supplementary Materials). Quantification of the gene relative abundance in each fish sample is presented in Table S7 (Supplementary Materials). All taxonomic relative abundance at levels from phylum to species is summarized in Table S8 (Supplementary Materials).

**Figure 4:**
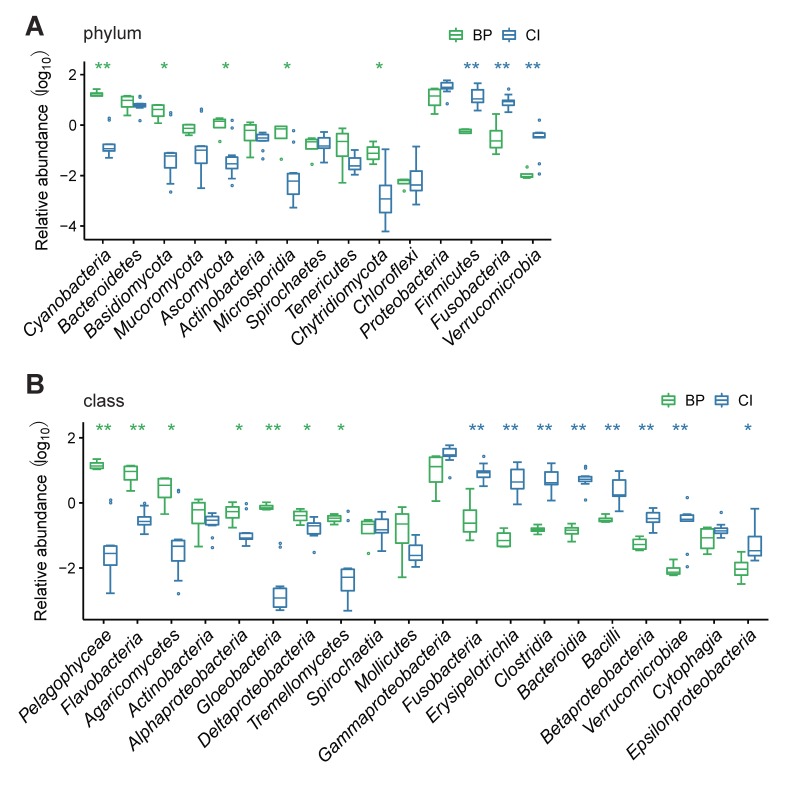
Top abundant microbiota at the phylum (**A**) and class (**B**) levels in herbivorous blue-spotted mudskipper (BP) and their composition in comparison with grass carp (CI). Significance: * 0.01 < *p* < 0.05, ** *p* < 0.01.

Significant differences in taxonomic composition and COG annotation were compared between BP and grass carp (Tables S9 and S10, Supplementary Materials), since both are herbivorous and had more than three GI samples. It was revealed that Cyanobacteria (17.96%) was the most abundant phylum in mudskipper GI (Figure 4A), which is consistent with our OTU data. Proteobacteria and Bacteroidetes were the core phyla in the five fish species, with a high richness (Table S8, Supplementary Materials). However, those phyla with high abundance in grass carp were at dramatically lower levels in BP, including Firmicutes (17.32% vs. 0.58%), Fusobacteria (10.06% vs. 0.83%), and Verrucomicrobia (0.44% vs. 0.01%). Pelagophyceae (15.1%) was determined to be the most widely distributed class in BP, while with a significant lower abundance in grass carp (0.24%). Gammaproteobacteria were largely present in both BP (15%) and grass carp (34%, Figure 4B), as well as the other three fish species. Notably, genes from Flavobacteriia still occupied a dominant position in the GI of BP (8.99%, vs. 0.38% in grass carp).

### 3.5. Functional Classifications

**Figure 5:**
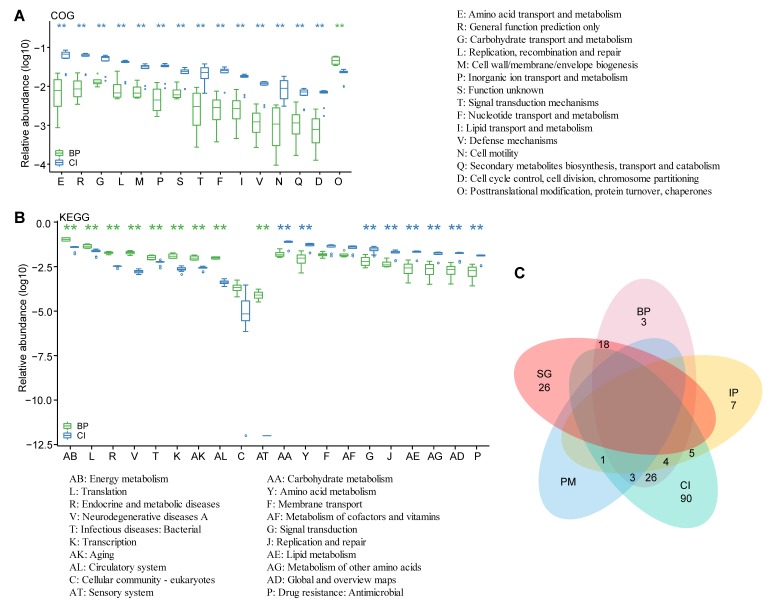
Differential analyses of functional genes. Significant differences in COG (**A**) and KEGG pathways (**B**) were determined between BP and grass carp (CI); (**C**) Venn diagram demonstrating overlaps in bacteriocin-related genes among the examined five common fish species, including blue-spotted mudskipper (BP), giant-fin mudskipper (PM), grass carp (CI), channel catfish (IP), and golden rabbitfish (SG). Significance: * 0.01 < *p* < 0.05, ** *p* < 0.01.

We assigned several important genes to known functional classifications based on both COG and KEGG annotations. Significant difference analysis between BP and grass carp on COG terms revealed that “posttranslational modification, protein turnover, chaperones” was the only category where BP outnumbered grass carp (Figure 5A), suggesting that the BP GI may have more critical components for cell signaling. As for the KEGG pathway terms (Figure 5B), there were many items with significantly different abundance between the two fish species. Genes related to energy metabolism in the GI of BP occupied a dominant position, whereas carbohydrate metabolism did so in grass carp. Some drug resistance genes were enriched in the GI of grass carp, implying that they may have been affected by exogenous probiotics or food-derived additives.

Bacteriocin-related genes were identified in the metagenomes, suggesting their potential immunological functions in the fish GI (Table S10, Supplementary Materials). A total of 183 related genes were annotated; among them, 129, 54, 44, 17, and four genes were confirmed in grass carp, BP, golden rabbitfish, channel catfish, and PM, respectively. Some of them existed in several fish species, while some of them were specific to only one fish species (for example, there was no overlap between BP and PM; see more details in Figure 5C). Many genes specific to BP and grass carp belonged to *Aeromonas* and *Shewanella*, in which *A. veronii* had a rather high level in grass carp. Three genes unique to BP, produced by *Shewanella* sp. MR-4 and *Vibrio furnissii*, were related to colicin transportation. Genes shared by channel catfish, grass carp, and PM were associated with microcin C from *Plesiomonas shigelloides*, which had a high level in channel catfish and PM. Genes commonly found in BP and golden rabbitfish mostly came from *Vibrio vulnificus*, which had an extremely high level in golden rabbitfish. As we know, most of these bacteriocin-related genes are reported to participate in transportation and production of bacteriocins (such as microcin, colicin, and lantibiotics), suggesting their potential contribution to pathogen resistance, especially in grass carp and blue-spotted mudskipper. 

## 4. Discussion

### 4.1. GI Bacterial Taxa in Different Vertebrates

For the examined fish species with different feeding habits, we obtained 706 core microbiomes (Figure 2D); among them, an overlap of 118 core OTUs was observed in the 134 core OTUs of the five groups with different living habitats, suggesting that most shared OTUs are essential for vertebrates. Most phylum-level assignments belonged to Proteobacteria and Firmicutes, consistent with many previous studies [27,28]. Freshwater fishes had a relatively inferior richness and diversity of the GI bacteria; however, the PCA and NMDS analyses showed that they had a wider range of microbiota composition than the other groups. Tenericutes, usually identified in the GI communities of fishes, juvenile amphibians, and corals [29,30], accounted for 6.19% in freshwater fishes, while it occurred at very low levels in the other four groups. This is in contrast to a previous report where marine adult salmon was enriched with Tenericutes, especially for the genus *Mycoplasma* [31]. Increasing the amount of fish metagenomic data via the elevation of fish species or sample numbers for each species could potentially help to resolve these contradictions.

Although mudskippers are amphibious fish species, they have limited common features with reptiles or other amphibians. A dramatic enrichment of Cyanobacteria in mudskippers was confirmed by both 16S ribosomal DNA (rDNA) sequencing and metagenomic analysis. These autotrophic prokaryotes have capabilities for oxygen production and nitrogen fixation, which may reveal their functions in mudskipper GIs. These characteristics are closely related to their aquatic to terrestrial habitats [32], which may imply the significance of GI microbiota to aerial gaseous exchange and water retention in mudskippers. It was also reported that these bacteria are able to synthesize vitamin B_12_ or have symbioses with B_12_-producing bacteria [33]. Some researchers believe that they are transient or allochthonous microorganisms from foods [34]. Their existence in BP may be not entirely associated with their food resources; however, it is potentially useful for energy production. This is consistent with the KEGG pathway enrichment analysis, and omnivorous PM also had a considerable quantity of Cyanobacteria (Table S4, Supplementary Materials). 

Grass carp was determined to have a high abundance of Cyanobacteria due to its feeding habits in a previous study [35]; however, we obtained different results in our 10 grass carp samples. In addition, we observed that *Cetobacterium somerae*, an indigenous bacterium with vitamin B_12_-producing ability in freshwater fishes [36,37], widely existed in freshwater fishes (20.74%), mudskippers (10.26%), and amphibians (2.19%) (Table S5, Supplementary Materials). Meanwhile, it was one of the core microbiota in the three examined fish groups with different feeding habits, suggesting its essential role for GI microbial functions. We also identified that the phylum Flavobacteriia, widely considered as an opportunistic pathogens for serious diseases in both farmed and wild fishes [38], appeared much more frequently in BP than in other fishes. Flavobacteriia have the remarkable ability to transform higher-molecular-weight compounds into lower molecules, and uptake hydrogen gas that is produced during nitrogen gas fixation [39]. Their high abundance in coastal marine biofilms is another indicator of their important functions [40]. Their roles in mudskippers are of interest to be further investigated.

Amphibians were found to possess several higher GI contents of phyla than the other four vertebrate groups, such as *Synergistetes* (9.17%), *Verrucomicrobia* (7.63%), *Deferribacteres* (2.79%), and *Planctomycete* (1.99%). *Verrucomicrobia* is widespread in marine environments [41]; however, our data in this study determined its low abundance in fishes with both freshwater and marine habitats as previously reported [31] and indicated a wider distribution. There were many differences among the five groups in this study, and most top abundant genera in the marine fish group were seldom present in the other four groups, such as *Vibrio* (12.93%), *Photobacterium* (12.44%), *Desulfovibrio* (5.05%), *Arcobacter* (2.58%), *Brevinema* (2.33%), *Halomonas* (2.03%), *Aliivibrio* (1.57%), *Mycobacterium* (1.46%), *Bacillus* (1.3%), and *Labrenzia* (0.98%). As a salt-tolerant genus usually present in marine environments, *Shewanella* in mudskippers (3.54%) presented an equally high level to marine fishes (3.87%). Within this genus, one of the top 10 abundant species, *S. benthica*, in the marine group (0.1%) was found at a higher level than in mudskippers (0.0003%), while it was not detectable in other groups; however, one of the top 18 abundant species, *S. hanedai*, was unique to marine fishes (0.05%). Their ability to reduce or degrade various substances makes them valuable tools for bioremediation, indicating great metabolic versatility in the GI tract of marine organisms [42,43].

The abovementioned genera *Vibrio* and *Photobacterium* widely exist in saltwater, and *Desulfovibrio* is commonly found in eutrophic aquatic environments. *V. rumoiensis*, reported to exhibit high catalase activity [44], was highly enriched in marine fishes (3.95%) over mudskippers (1.37%), indicating important roles of enteric bacteria in oxidative stress response. *Photobacterium*, a halophilic pathogen among marine animals and humans [45], was much more abundant in marine fishes (12.44%) and carnivores (7.16%) based on our present work. *P. damselae* is a core gut microflora in sharks [8], which is also consistent with our present report of high abundance in marine fishes (7.08%). The second abundant species *P. angustum* in the marine fish group accounted for 5.29%, while it represented only 0.02% in mudskippers. Interestingly, some members of *Photobacterium* are symbiotic bacteria aiding with chitin digestion [46], possibly helping fishes to digest crustaceans. There were some other well-known marine salt-tolerant microorganisms [47] present in the examined fishes, such as *Marinobacter* in marine fishes, and *Idiomarina*, *Ruegeria*, and *Alteromonas* in mudskippers and marine fishes. The extremely salt-tolerant bacteria *Halomonas* [48], with abundance only in the marine fish group, is also worthy of an in-depth investigation. Nevertheless, the sophisticated networks and various complex relationships among microbes in fish GIs are far from being elucidated.

### 4.2. Factors for Shaping the GI Microbiome

The highly diverse GI microbiota composition in various host individuals gives rise to the community structure of any species with high heterogeneity. Many factors were reported to influence the formation of GI microbes, and these commensal bacteria have various effects on the hosts [5,49,50]. In the human GI microbiota, the Firmicutes/Bacteroidetes ratio is an indicator of the growth rate and adipose accumulation [51]. A higher ratio in transgenic carp also led to faster growth [52]. These results seem to demonstrate the importance of the relative proportion of microbial taxa. In our present work, the Firmicutes/Bacteroidetes ratios in terrestrial animals were lower than those in other four groups, and the terrestrial group was the only one with a higher abundance of Bacteroidetes than Firmicutes (Figure 3A). However, in the two mudskippers with different living habitats, it seems that the more terrestrial PM showed a higher Firmicutes/Bacteroidetes ratio than the less terrestrial BP in the metagenome taxonomic analysis (Table S8, Supplementary Materials), although confirmation is necessary following studies with large-scale samples.

The covariation of environmental parameters [53], gut physiology [54], different development stages [55], weight [56], diet [57], and host genotype [58] contributes to the constitution of gut microbial communities. A previous comparison of the stomach microbiome between river-dwelling and cave-restricted Mexican tetra (*Astyanax mexicanus*) populations concluded that the microbiota is associated with water parameters but not the contrasting habitats [59]. A biogeography analysis of the Atlantic salmon gut microbiome also showed that its life-cycle stage strongly defined microbial assemblages but not geography [31]. On the other hand, a distinct microbiome structure was characterized between laboratory and wild common carps, i.e., lake- and river-dwelling common carps, while diet did not exert much effect on the gut microbiota [34]. A previous study showed that sex and feeding strategies had different impacts on the GI microbiota communities of BP and PM [60], and this report confirmed the high abundance of Cyanobacteria in female PM, albeit with slight differences from our results in the dominant genera. Convergent evolutionary ties between the symbiotic bacteria of fishes and mammals [28] reflect that fishes harbor more complicated gut communities, which is consistent with our present comparisons between the compositions in marine fishes and terrestrial vertebrates (Figure 3A). 

A comparison between the GI microbiomes of 18 primate species [61] showed that gut microbial composition and function are more affected by differences in host physiology or phylogeny than variances in host geographic location or actual dietary intake, indicating that the gut microbiome plasticity should consider host physiology, nutritional strategies, and the emergence of dietary niches. A 28-case-control study in the human gut microbiome [62] argued that many associated pathogenic microbes are not likely disease-specific but shared by healthy people and patients, suggesting that the combination of several clinical diseases and comparisons of published data is indispensable for the identification of marker bacteria. Here, the classifications based on living habitats and feeding habits differentiated the core and unique taxa in various vertebrate species; however, unified standards and big databases should be established for better utilization in future researches.

In summary, many deterministic and stochastic processes shape the complexity of GI microbiomes. In this study, dramatic differences were determined between vertebrate groups with various habitats or feeding habits, although our current data are somewhat preliminary. Many potential biases would exist in the pooling of different datasets as many parameters cause individual variations and result in uncertainty in the conclusions. However, a big-data-based analysis is necessary for understanding microbiome differences among species with varied kinships. Therefore, meticulous studies of every GI microbiome species should be realized for the accumulation of comprehensive knowledge about core microbes that may benefit target hosts. 

### 4.3. Functional Potentiality of Metagenomes

The use of metagenomes facilitates the characterization of functional genes that play important roles in hosts, especially for gene clusters of microbial natural products and secondary metabolites. A distinctive functional repertoire was built in the human gut microbiota, which could identify biosynthetic gene clusters to explain their elusive nature [63]. A causal effect of the gut microbiome on metabolic traits such as the production of short-chain fatty acids was found to be critical to human metabolic diseases [64]. In aquaculture, the biosynthetic potential of diverse microorganisms was underestimated for a long time [65]. Lactic acid bacteria from several finfish species are regarded as a potential resource of new antibiotics and pharmaceutical compounds [66]. Natural products from fish GI bacteria are valuable resources for the development of probiotics, especially for bacteriocins that are the best alternatives for antibiotics. 

In the present study, the significant abundance of bacteriocin-related genes in the examined grass carp suggested potential influence by artificial factors in aquaculture. In the metagenome analysis of two herbivorous fishes in this study, the lower amount of BP sequencing data limited its comparison with its grass carp counterpart, since there were many unclassified taxa that may indicate novel bacterial types and the uniqueness of this amphibious fish. Although the numbers of bacteriocin-related genes in PM, channel catfish, and golden rabbitfish were limited as a result of difficulties in GI sample collection, these genetic resources could also provide useful information for the development of probiotics [67] and characterization of antibiotic resistance genes for practical aquaculture [68]. The antibiotic gene number in GI microbial communities is also influenced by many factors outside of data quantity. For example, antibiotic activity-related microbial genes in the Asian bass gut microbiome were significantly enriched under starvation [69]. The bacterial taxa harboring antibiotic resistance genes in the gut microbiome of *Piaractus mesopotamicus* were elevated with oral administration of antibiotics [70]. *Vibrio furnissii* in BP is considered as a potential pathogen for European eel [71], and many *Vibrio* strains were identified in diseased shrimps [72]. *Aeromonas veronii* dominant in grass carp is a beneficial symbiont of leeches [73], but may be a causative epizootic ulcerative syndrome agent for fishes in Bangladesh [74]. Actually, diverse virulence factors in *Aeromonas* were recognized in fish diseases [75,76]. *Plesiomonas shigelloides* in channel catfish and PM was reported to cause subcutaneous hemorrhagic ulcers, as well as kidney and liver necrosis in silver carp [77] (leading to up to a 60% mortality rate) and intestinal diseases in humans [78], as it is a cytotoxic enterotoxin producer [79]. *Vibrio vulnificus* in BP and golden rabbitfish is pathogenic to tilapia [80], shrimp [81], eels, and humans [82], and it was reported as the most infectious and lethal zoonotic *Vibrio*. Normally, the bacteriocin-producing bacteria are in symbiosis with their hosts, but they can be lethal to other animals under particular conditions [83]. These data may provide valuable instructions for aquaculture management.

In the case of KEGG pathways, carbohydrate metabolism and amino-acid metabolism were the top two differentially abundant pathways in grass carp, while the GI microbiota of BP was more involved in energy metabolism and translation, suggesting distinct metabolic affinities between the two herbivorous fishes. As for COG terms, we observed only one particularly abundant gene in BP GI, which was associated with protein modification and chaperones, indicating that there is a more complex molecular network. The enrichment of various genes in grass carp GI suggests a variety of functions for its microorganisms. A large number of unknown bacteria lead to unclear functioning features in the host GI, thus raising the importance of more in-depth metagenome studies. This will be indispensable to uncover the mysterious relationships between hosts and GI microbiota. 

## 5. Conclusions

In this study, we performed the first comprehensive investigation into the GI microbiota of amphibious mudskippers, and established comparisons with other freshwater and marine fishes, amphibians, and land-dwelling vertebrates. In general, we observed that Proteobacteria, Firmicute, and Bacteroidetes were the most abundant core microbiomes. The first gene catalogue of the mudskipper GI microbiome was also reported, and comparisons of taxonomy and differential functional genes were examined. How the diet, genetic background, and environment alter the GI microbiota of fishes needs to be comprehensively studied and combined with big data for various species, as briefly discussed in Section 4. Further research into these microorganisms and their functions in the host GI are necessary in order to establish any causality in fish disease pathogenesis and to develop potential probiotics for practical aquaculture.

## Supplementary Materials

The following are available online at https://www.mdpi.com/2076-2615/9/9/660/s1: Table S1: Summary of the achieved data from 16S rDNA amplicon and metagenome sequencing; Table S2: OTU abundance, taxonomy, and comparisons among different vertebrate groups; Table S3: Alpha diversity values of all samples and comparison among groups; Table S4: Taxonomy relative abundance at level from phylum to species in each sample of 16S rDNA amplicon sequencing; Table S5: Differential analysis of taxonomy relative abundance at the levels from phylum to species among various groups with different habitats and feeding habits; Table S6: Annotation of 155,217 assembled genes and taxonomy of 132,183 genes in the metagenome sequenced samples; Table S7: Relative abundance of genes in each metagenome sequenced sample; Table S8: Taxonomy relative abundance at the levels from phylum to species in each metagenome sequenced sample; Table S9: Significant analysis of taxonomic relative abundance at the levels from phylum to species between herbivorous BP and grass carp; Table S10: Significant analysis of functional pathways (via COG and KEGG) between BP and grass carp, and identification of bacteriocin-related genes in five fish species.
